# The Spectrum of Care for Aortic Disease

**DOI:** 10.14797/mdcvj.1217

**Published:** 2023-03-07

**Authors:** Alan B. Lumsden

**Affiliations:** 1 Methodist DeBakey Heart & Vascular Center, Houston Methodist, Houston, Texas, US

**Keywords:** aortic disease, endograft, graft explantation, abdominal aortic aneurysm

Aortic diseases have varied pathologies and, increasingly, new options for treatment. The causes of aortic disease may vary from congenital or genetic conditions to infection, traumatic accidents, degenerative or inflammatory disorders. Environmental influences clearly contribute to increased occurrence, with known risk factors including hypertension, aging, smoking, and trauma. While preventive and pharmacological interventions have been explored in recent years, surgery or Endovascular intervention continues as the recommended solution when treating most cases. This issue of the *Methodist DeBakey Cardiovascular Journal* explores the major types of aortic disease and their various options for treatment and repair.

We open with a review of dynamic imaging of aortic pathologies by Drs. Dipan J. Shah, Alan B. Lumsden, Peter Osztrogonacz, and colleagues, who note that today’s vascular surgeons can choose from a wide array of imaging modalities to evaluate different aortic pathologies. Despite the fact that newer dynamic imaging techniques provide time-resolved information in various aortic pathologies compared to the standard imaging modalities of vascular ultrasound and aortography, these newer techniques have not seen widespread adoption. To help readers decipher the role of emerging modalities, the authors provide an overview of dynamic imaging techniques for aortic pathologies and describe various dynamic computed tomography and magnetic resonance imaging protocols, clinical applications, and potential future directions.

Next, we explore the topic of branched and fenestrated aortic endovascular grafts with Dr. Marvin D. Atkins and coauthor Aidan D. Atkins. Although improvements in morbidity and mortality have made endovascular repair of abdominal and descending thoracic aortic aneurysms the standard of care, late durability is problematic due to persistent endoleaks that may lead to aneurysm expansion and eventual rupture. The authors explore the current state of branched, fenestrated, and physician-modified endografts used in complex aortic pathologies—including early results of such technologies that show at least similar short-term outcomes compared with open repair—as well as issues such as late branch endograft failure and acute periprocedural stroke.

From here, Drs. Valeria Duarte, Michael Singh, and Raman Yousefzai discuss the increase in genetically triggered thoracic aortic disease. Up to 25% of patients with thoracic aortic disease have an underlying genetic predisposition, which makes diagnosis critical from both a medical management perspective and to identify at-risk family members. The authors explain the genetic etiology of thoracic aortic disease, identify aortic diseases associated with genetic syndromes, review the recent ACC/AHA guidelines, and discuss specific testing protocols for patients predisposed with heritable thoracic aortic disease.

Authors Michael Reardon, Marvin Atkins, and Aidan Atkins then delve into endovascular management of the ascending aorta. Thoracic endovascular aortic stent graft repair (TEVAR) has revolutionized the management of descending aortic pathologies, showing significant improvements in mortality, morbidity, and recovery time. However, devices designed specifically for the ascending aorta are desperately needed for those patients unfit for open surgical repair. The authors update readers on the unique challenges of endovascular management of the ascending aorta and look at the future technologies that will define this space.

The dramatic increase in the number of aneurysms treated with abdominal aortic endografts has spurred more interest in graft explantation. Open procedures can be particularly challenging, especially in patients with nonremedial type 1 endoleaks or graft infections. With up to 70% of primary repairs performed with an abdominal aortic endograft, an overwhelming number of explants continue to be performed in the abdomen. In the article titled “Explant of the Aortic Endograft: Today’s Solutions, Tomorrow’s Problems,” I address some of the technical challenges and management of these patients and focus on practical challenges associated with removing these devices.

From here, Drs. Anthony Estrera, Akiko Tanaka, and colleagues review open treatments for thoracoabdominal aortic aneurysm repair (TAAA), for which Michael E. DeBakey and colleagues developed a Dacron graft to shunt between the descending thoracic aorta and infrarenal abdominal aorta to minimize visceral and renal ischemia. The current standard practice of TAAA repair includes myriad techniques, such as left heart bypass with mild hypothermia or cardiopulmonary bypass with moderate/deep hypothermia, among others, and the authors explore these techniques in detail. They also explain how their use of distal aortic perfusion and cerebrospinal fluid drainage combined with moderate passive hypothermia has reduced the overall spinal cord ischemia rate after extent I TAAA.

Drs. John F. Eidt and Javier Vasquez then shift focus to the changing management of type B aortic dissections (TBAD). Despite contemporary advances in medical and surgical care, acute aortic dissection remains a highly lethal and morbid condition, with almost one-third of patients never reaching the hospital. The authors explore four significant trends in the management of type B aortic dissection, including (1) the adoption of a new classification system that resolves deficiencies of the DeBakey and Stanford systems; (2) recognition that thoracic endovascular aortic repair (TEVAR) fails to effectively prevent aneurysmal degeneration in the untreated aortic segments; (3) increasing use of TEVAR in uncomplicated TBAD despite the absence of definitive proof of efficacy; and (4) the emergence of a variety of techniques and devices designed to improve the long-term outcomes of TBAD.

We then step back to look at the medical management of aortic disease. While surgery is the cornerstone of management for most aortic conditions, medical therapy is now an important adjunctive therapy in most if not all aortic patients. A preemptive diagnostic approach with a multidisciplinary team and shared decision-making has led to improved clinical outcomes. Control of blood pressure, cholesterol, lifestyle factors, and comorbidities are all important and meaningful targets to optimize outcomes in aortic disease patients. In their review, Drs. Maan Malahfji and Mujtaba Saeed review the role and evidence behind medical management of patients with aortic disease.

Ruptured abdominal aortic aneurysm is an acute aortic condition that requires immediate action and appropriate continuity of care to optimize patient outcomes. A systematic standardized protocol-driven approach is essential to improve the management of aortic emergencies as well as perioperative morbidity, mortality, and long-term survival. In their review, authors Maham Rahimi, Peter J. Osztrogonacz, Vy C. Dang, and colleagues summarize the internal protocol of the Houston Methodist Hospital Acute Aortic Treatment Center to provide guidance to vascular surgeons in training and also to low-volume aortic centers, following the five phases of surgical care outlined by the American College of Surgeons (ACS): preoperative, perioperative, intraoperative, postoperative, and post-discharge.

The quality of any cardiovascular program depends in large part on the competency and care of its nursing staff. Having specialty trained and certified cardiovascular nurses has been shown to help decrease delays in cardiac referral and treatment, reduce hospital stays in patients with cardiac disease, and prevent rehospitalization. Against the backdrop of an ongoing nurse shortage, healthcare organizations need strategic plans to support, build, and retain a strong team of CV nurses. Gail M. Vozzella, DNP, RN, NEA-BC, and Michelle C. Hehman, PhD, RN, close this issue describing the numerous forces driving the current nursing shortage and the impact of the coronavirus-19 pandemic on nurse job satisfaction and turnover. They also present a reinvented model of nursing care as a framework for healthcare organizations to address nurse staffing challenges.

The articles presented here are shared in the spirit of offering useful insights and approaches for treating aortic diseases and repairing the damage they have caused. To learn more about aortic disease treatment and see additional video clips of aortic procedures, I include links to the extensive video library of DeBakey Cardiovascular Education. More videos, in addition to those shared in the articles included here, can be found on the DeBakey Cardiovascular Education YouTube channel at https://www.youtube.com/c/HoustonMethodistDeBakeyCVEducation.

## Guest Editor Biography

The editors of the *Methodist DeBakey Cardiovascular Journal* express our appreciation to Dr. Alan B. Lumsden for his knowledge and insight in developing this issue on aortic disease.

## Alan B. Lumsden, MD

**Figure:**
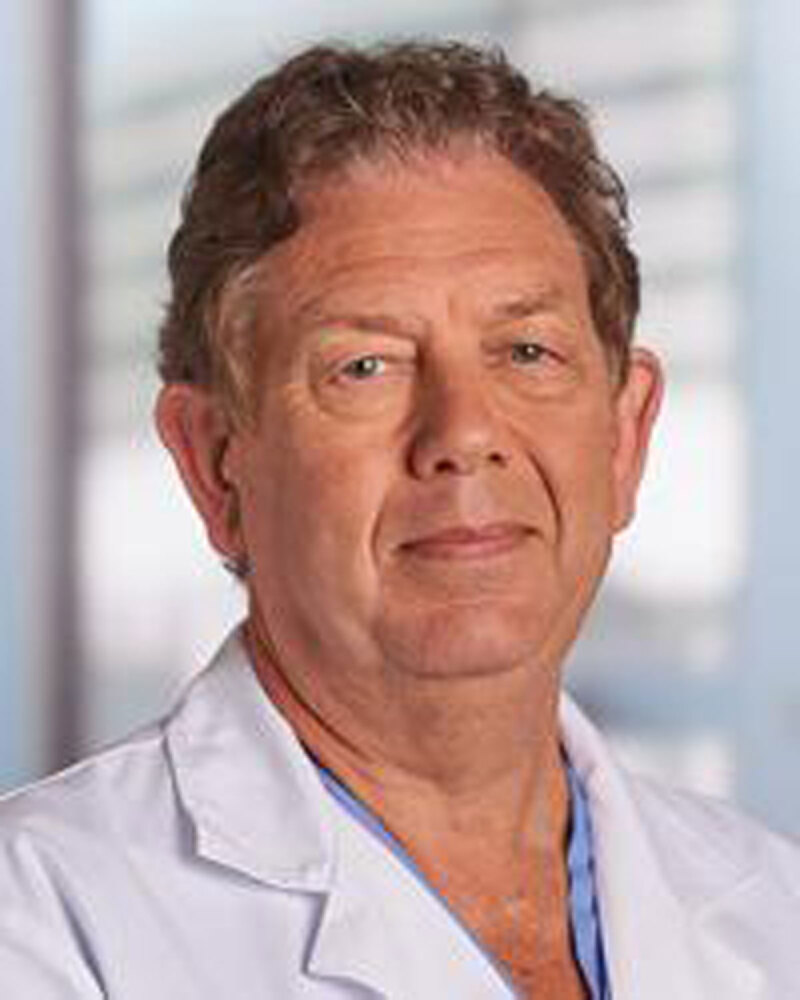


Dr. Lumsden’s academic career began in 1989 at Emory University in Atlanta, Georgia, where he completed his postdoctoral training and served as a Collaborative Scientist in the Division of Pathobiology and Immunology at the renowned Yerkes Primate Center. He remained at Emory for several years, rising to the rank of associate professor and chief of the Division of Vascular Surgery. In 2002, Dr. Lumsden joined the faculty at the Baylor College of Medicine in Houston, Texas, as professor and chief of the Division of Vascular Surgery and Endovascular Therapy. He assumed his current positions at Houston Methodist in 2008.

Dr. Lumsden has developed an international reputation as a leader in the field of endovascular surgery. He has clinical and research expertise in stent graft treatment of thoracic and abdominal aortic aneurysms, stenting and endarterectomy in carotid arterial disease, renovascular hypertension, aortoiliac occlusive disease, and mesenteric vascular and minimally invasive therapy in venous disease. Dr. Lumsden also conducts FDA-mandated training for surgeons nationwide in a carotid stenting simulator housed at the Houston Methodist DeBakey Heart & Vascular Center.

Dr. Lumsden’s research interests include developing novel minimally invasive methods of therapy and preventing restenosis. His research has been funded by the National Institutes of Health, and he has contributed more than 200 papers to the medical literature, as well as numerous abstracts, books, and book chapters.

